# Crosstalk between circRNAs and the PI3K/AKT signaling pathway in cancer progression

**DOI:** 10.1038/s41392-021-00788-w

**Published:** 2021-11-24

**Authors:** Chen Xue, Ganglei Li, Juan Lu, Lanjuan Li

**Affiliations:** 1 State Key Laboratory for Diagnosis and Treatment of Infectious Diseases, National Clinical Research Center for Infectious Diseases, Collaborative Innovation Center for Diagnosis and Treatment of Infectious Diseases, The First Affiliated Hospital, College of Medicine, Zhejiang University, Hangzhou, 310003 China; 2 Department of Neurosurgery, The First Affiliated Hospital, College of Medicine, Zhejiang University, Hangzhou, 310003 China

**Keywords:** Cancer therapy, Oncogenes

## Abstract

Circular RNAs (circRNAs), covalently closed noncoding RNAs, are widely expressed in eukaryotes and viruses. They can function by regulating target gene expression, linear RNA transcription and protein generation. The phosphoinositide 3-kinase (PI3K)/AKT signaling pathway plays key roles in many biological and cellular processes, such as cell proliferation, growth, invasion, migration, and angiogenesis. It also plays a pivotal role in cancer progression. Emerging data suggest that the circRNA/PI3K/AKT axis modulates the expression of cancer-associated genes and thus regulates tumor progression. Aberrant regulation of the expression of circRNAs in the circRNA/PI3K/AKT axis is significantly associated with clinicopathological characteristics and plays an important role in the regulation of biological functions. In this review, we summarized the expression and biological functions of PI3K-AKT-related circRNAs in vitro and in vivo and assessed their associations with clinicopathological characteristics. We also further discussed the important role of circRNAs in the diagnosis, prognostication, and treatment of cancers.

## Introduction

The complexity of cancer and the variability of its clinical features are derived from its complex etiology, involving DNA, RNA, protein, and other factors.^1–3^ Cancer has become an important public health concern affecting people’s lives.^4–6^ In the past 10 years, the number of studies on cancer has increased rapidly, providing many novel clues for the treatment of cancer.^7,8^ The emergence of targeted therapy and immunotherapy has greatly improved the survival rate of cancer patients.^9,10^ However, cancer treatment remains a major scientific challenge.

Circular RNAs (circRNAs), a newly discovered type of noncoding RNA, have a covalently closed structure and high stability.^11–13^ CircRNAs are mainly formed by pre-mRNA a back-splicing and are widely expressed in eukaryotes and viruses.^14,15^ The regulatory role of circRNAs in physiological processes is still not very clear.^16^ However, accumulating evidence indicates that circRNAs are significantly associated with many diseases and play an important role in the occurrence and development of cancer. A common circRNA-mediated mechanism is that circRNAs act as competitive endogenous RNAs (ceRNAs) of microRNAs (miRNAs) in tumor progression. Circ101237 facilitates the expression of MAPK1 to suppress tumor progression by sponging miR-490-3p in non-small cell lung cancer (NSCLC).^17^ CircRNA also regulates cancer development and progression by interacting with protein. CircRNA cIARS suppresses cell autophagy via binding with RBP ALKBH5.^18^

**Fig. 1:**
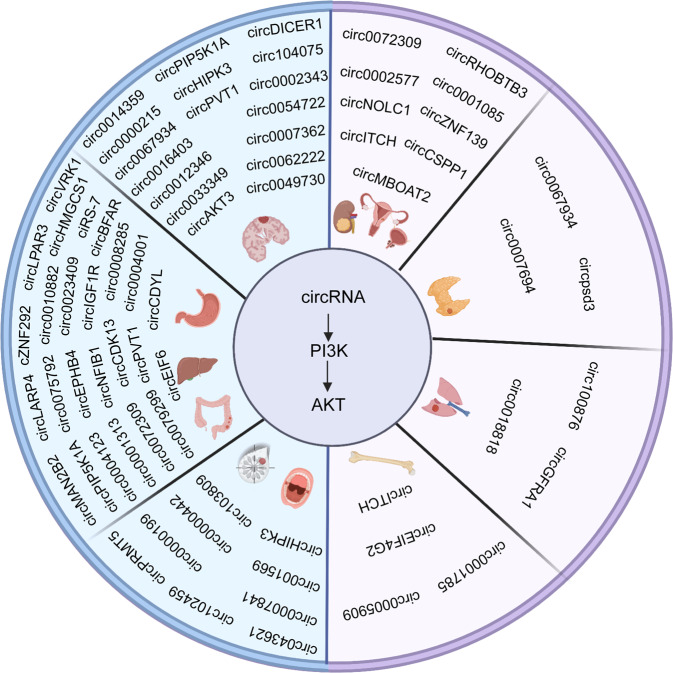
CircRNAs interact with the PI3K/AKT pathway to regulate cancer progression. Image created with BioRender (https://biorender.com/)

Phosphoinositide 3-kinase (PI3K), a member of the lipid kinase family, is an important regulator of signaling and intracellular vesicular trafficking.^19^ Several studies have found that the PI3K/AKT pathway is aberrantly activated in cancer^20–22^ and controls core cellular functions, such as proliferation and survival.^23,24^ The PI3K/AKT pathway plays a pivotal role in the progression of cancer. Clinical trials targeting PI3K have also attracted increasing attention.^25,26^ Emerging evidence suggests that circRNAs interact with the PI3K/AKT pathway to regulate cancer progression. Importantly, circRNAs related to the PI3K/AKT pathway have become potential targets in the treatment of cancer. In this review, we summarized the current studies of the role of crosstalk between circRNAs and the PI3K/AKT pathway in the initiation and progression of cancer (Fig. 1). We also presented the clinical applications of PI3K/AKT-related circRNAs in patients with cancer.

## The PI3K/AKT signaling pathway in tumorigenesis

### PI3K

Phosphoinositide 3-kinase (PI3K), a member of the lipid kinase family,^27,28^ was first identified 3 decades ago.^29^ It can be divided into 3 types (class I–III) in mammals.^19,30,31^ Class I PI3Ks have gained much attention in the cancer-related field. PI3K is composed of one catalytic (p110) domain and one regulatory (p85) domain.^32,33^ p85, which contains the Src homology 2 (SH2) and SH3 protein-binding domains,^34,35^ can interact with target proteins with corresponding binding sites. The activation of PI3K mainly involves the binding of the substrate near the inner side of the plasma membrane.^36,37^ PI3K can be activated in two ways. One is that PI3K interacts with connexin or growth factor receptors with phosphorylated tyrosine residues, and then induces a conformational change of dimer.^38–40^ It also can be activated by the direct binding of p110 and Ras.^41–43^

**Fig. 2:**
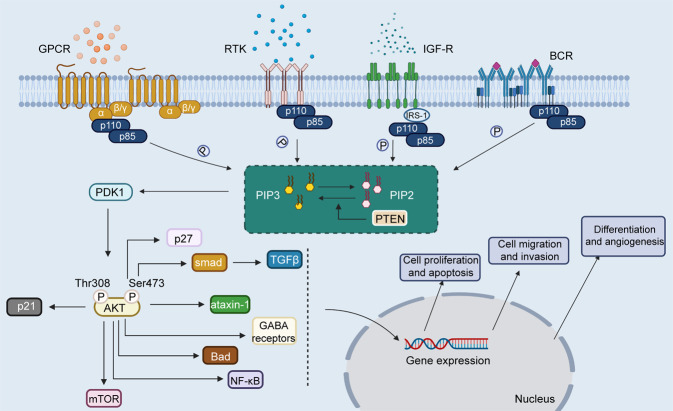
The activation process of PI3K/AKT signaling pathway. PI3K, composed of one catalytic (p110) domain and one regulatory (p85), can be activated by G-protein coupled receptor, RTK, IGF-R, and B-cell receptor. Activated PI3K facilitates the conversion of PIP2 to PIP3. PIP3 activates PDK1, and then PDK1 phosphorylates AKT at Thr308. AKT can be also phosphorylated, and activated by PDK2 at Ser473. Activated AKT can regulate mangy cellular biological functions by interacting with numerous downstream signaling molecules, such as p21, p27, TGFβ, ataxin-1, GABA receptors, Bad, NF-κB, and mTOR. Image created with BioRender (https://biorender.com/)

PI3K can be activated by multiple growth factors and signaling complexes, such as G-protein coupled receptors, B-cell receptors, vascular endothelial growth factor (VEGF), fibroblast growth factor (FGF), insulin and receptor tyrosine kinases (RTKs) (Fig. 2).^20,44–48^ These factors induce autophosphorylation through the activation of RTKs and then activate PI3K.^49^ The p85 subunit provides docking sites for autophosphorylation. In some cases, this process is mediated by the recruitment of adapter proteins. For example, the insulin receptor activates PI3K via insulin receptor substrate-1 (IRS-1).^50,51^ Activated PI3K increases the conversion of PIP2 to PIP3, which activates PDK1 and AKT.^52,53^ However, AKT is not the only target molecule of PI3K. PI3K regulates multiple signaling pathways by interacting with BTK, PDK1, and Rac.^54^

### AKT

AKT, also called protein kinase B (PKB),^55,56^ is the cellular homolog of the oncogene v-Akt. AKT is a serine/threonine kinase that belongs to the AGC kinase family.^57–59^ There are three different AKT isoforms (AKT1, AKT2, and AKT3), which are widely expressed in most human tissues.^60–62^ AKT can link the interaction between receptors and PI3K to cellular anabolic pathways. AKT acts as a central regulator of cellular metabolism downstream of insulin signaling that is responsible for the regulation of glucose metabolism.^63,64^ In vivo experiments support that AKT2 plays a key role in the regulation of glucose metabolism.^65,66^ Researchers have found that germline mutations of AKT occur during the tumorigenesis and progression of some cancer.^67,68^

AKT plays a key role in multiple cellular processes, such as cell survival, proliferation, migration, apoptosis, and angiogenesis.^69–72^ AKT prevents TSC1/TSC2 complex formation and activates mTOR pathway, thereby regulating cell growth.^73–75^ It also regulates the expression of cyclin D1 and p53 to affect the cell cycle or the proliferation of various cell types through interacting with CDK inhibitors including p21 and p27.^76^ AKT boosts cell survival via inactivating the pro-apoptotic factors Bad and the transcription factor of the Forkhead (FKHR) family.^77^ The expression levels of GABA receptors and ataxin-1 are also regulated by AKT.^78,79^ Some studies observed that AKT regulates the TGFβ signaling pathway by binding with Smad.^80^ The present findings show that AKT is an important target for the treatment of cancer, diabetes, stroke, and neurodegenerative diseases.^81–83^

### The activation of PI3K/AKT pathway

The PI3K/AKT signaling pathway plays key role in many biological and cellular functions.^84,85^ We have already elaborated on the activation of PI3K when introducing PI3K. The inositol ring of PI has five potential phosphorylation sites. PI3K activation could catalyze the phosphorylation of phosphatidylinositol (PI) at the 3′-position of the inositol ring.^86^ The phosphorylated products have a critical influence on cellular functions. PIP3 could enhance cell migration,^87^ and PI 3,4-bisphosphate regulates B cell activation and insulin sensitivity.^88^ AKT and PDK1, which contain PH domains can bind to PIP3. PIP3 activates PDK1,^89^ and then PDK1 phosphorylates AKT at Thr308.^90,91^ AKT can be also phosphorylated and activated by PDK2 at Ser473.^92,93^ Activated AKT regulates cell proliferation, differentiation, migration, and apoptosis by activating or inhibiting downstream target proteins, such as Bad,^94^ Caspase9,^95^ NF-κB,^96,97^ GSK-3,^98^ FKHR,^99,100^ p21,^101^ p53^102^ and FOXO1.^103,104^ Aberrant activation of PI3K/AKT pathway has been found in a variety of cancers,^105^ such as lung cancer,^106^ esophageal cancer,^107^ gastric cancer,^108^ breast cancer,^109^ laryngeal cancer,^110^ gallbladder cancer,^111^ and prostate cancer.^112^

PTEN is a widely mutated tumor suppressor gene that inhibits the oncogenic PI3K/AKT pathway.^113–115^ PTEN antagonizes the PI3K/Akt pathway by dephosphorylating PIP3 to PIP2,^116,117^ then induces changes in a variety of cellular biological functions.^118,119^ Carboxyl-terminal modulator protein (CTMP) could block the transmission of downstream signaling pathways by inhibiting AKT phosphorylation.^120,121^ PP2A has been found to dephosphorylate AKT-Thr308 and AKT-Ser473 to inhibit the activation of AKT.^122,123^

## CircRNAs and cancer

CircRNAs were initially found in RNA viruses at the end of the 20th century and were considered transcriptional background noise.^124–126^ With the application of high-throughput RNA sequencing and bioinformatics approaches, circRNAs have attracted much attention from researchers.^13,127,128^ CircRNAs, covalently closed noncoding RNAs, are widely expressed in eukaryotes and viruses.^11,129–131^ Linear pre-mRNAs generate circRNAs through exon skipping or back-splicing events.^132,133^ The circular form of circRNAs protects them from degradation by exonucleases, causing them to show greater stability.^11,12^ CircRNAs can function by regulating target gene expression, linear RNA transcription, and protein generation.^13,134,135^ Moreover, circRNAs are involved in the occurrence and development of several cancers.^129,136–139^ Different circRNAs play distinct roles in diverse cancer types. The circRNA cSMARCA5 has tumor-suppressive properties in the progression of hepatocellular carcinoma.^136^ However, circMAPK4 suppresses cell apoptosis by regulating specific pathways in gliomas.^140^

There are mainly four mechanisms by which circRNAs can act in cancer progression: miRNA sponging, protein binding, regulation of gene transcription, and regulation of protein translation. CircRNAs function as natural miRNA sponges that regulate miRNA activity.^141–143^ miRNAs are essential players in almost all carcinogenic processes.^144–146^ Increasing evidence suggests that circRNAs modulate cancer progression by regulating the expression of miRNA targets.^147–151^ For example, cTFRC facilitates tumor progression by sponging miR-107 in bladder carcinoma.^152^ In addition, circRNAs regulate cancer development and progression by directly modifying the transcription of related genes. Zhang et al.^153^ reported a novel class of intron-derived circRNAs that is widely distributed throughout the nucleus. Intron-derived circRNAs can interact with RNA polymerase II to enhance the transcription of its target genes.^154,155^ CircRNAs could also act as protein decoys, and regulate RNA-binding proteins (RBPs) activity by combining with RBPs.^156,157^ The expression of circZKSCAN1 attenuates HCC cell stemness by targeting RBP fragile X mental retardation protein.^158^ Moreover, some circRNAs containing the AUG start codon and IRES can control gene expression at the translational level.^159,160^ However, this effect has not yet been fully elucidated in cancer.

## The circRNA/PI3K/AKT axis in cancer

CircRNA plays a critical role in the initiation and development of human cancer.^161–165^ The studies on circRNA are changing our view of cancer genesis, progression, and treatment.^166,167^ CircRNAs alone may be insufficient for driving cancer progression. Similarly, traditional signaling pathways or signaling molecules alone may also be ineffective. Interestingly, studies have found that circRNAs are often interrelated with the PI3K/AKT signaling pathway. The PI3K/AKT signaling pathway plays key roles in many biological and cellular functions, such as cell proliferation, growth, invasion, migration, and angiogenesis.^85,168^ It also plays a pivotal role in the progression of cancer.^27,169,170^ Recently, a great deal of research regarding the interaction of circRNA and PI3K/AKT signaling pathways has attracted significant research interest. CircRNAs regulate cellular functions and control the occurrence and development of cancer via interactions with the PI3K/AKT pathway. Based on the current study, the mechanism/pattern of interaction between circRNA and PI3K/AKT pathway is primarily the ceRNA mechanism, which involves the activation or repression of downstream pathways by sponging miRNA. Research on the circRNA/PI3K/AKT axis is still in its infancy. With the deepening of research about the structure and function of circRNAs, the mechanism will add clarity regarding the circRNA/PI3K/AKT axis.

## Clinical features and cell biological functions related to the circRNA/PI3K/AKT axis

A large number of circRNAs have been found to be involved in the PI3K/AKT signaling pathway. The circRNA/PI3K/AKT axis modulates the expression of cancer-associated genes and thus regulates tumor progression. The circRNA/PI3K/AKT axis plays important role in the initiation and progression of several types of cancer. Current studies may lay the foundation for further research on the mechanisms of cancer progression and provide insights into circRNA-based clinical applications. In this section, we will summarize the expression, biological functions in vitro (Table 1), and associations with clinicopathological characteristics of circRNAs related to the PI3K/AKT signaling pathway (Table 2).

**Table 1:** Role and biological functions of circRNA/PI3K/AKT axis in cancer progression in vitro

Category	Type	CircRNA	Role	Function	Related genes; in vivo	Refs.
Digestive system neoplasms	Esophageal cancer	circLPAR3	Oncogene	Cell migration and invasion	miR-198, MET, RAS, MAPK, PI3K, and AKT	^171^
	Esophageal cancer	cZNF292		Cell viability, migration, invasion, and apoptosis	miR-206, AMPK, PI3K, and AKT	^174^
	Esophageal cancer	circVRK1	Tumor suppressor	Cell proliferation, migration, EMT, and radioresistance	miR-624-3p, PTEN, PI3K, and AKT	^172^
	Esophageal cancer	circLARP4	Tumor suppressor	Cell proliferation, migration, and apoptosis	miR-1323, PTEN, PI3K, and AKT	^173^
	Gastric cancer	circPIP5K1A	Oncogene	Cell proliferation, migration, invasion, and EMT	miR-671-5p, KRT80, PI3K, and AKT	^175^
	Gastric cancer	circ0010882	Oncogene	Cell proliferation, migration, invasion, and apoptosis	PI3K, Akt, and mTOR	^176^
	Gastric cancer	circ0023409	Oncogene	Cell viability, proliferation, migration, invasion, and apoptosis	miR-542-3p, IRS4, PI3K, and AKT	^177^
	Gastric cancer	ciRS-7	Oncogene		miR-7, PTEN, PI3K, and AKT	^178^
	Gastric cancer	circMAN2B2	Oncogene	Cell viability, cell survival, migration, and apoptosis	miR-145, PI3K, AKT, and JNK	^179^
	Gastric cancer	circPVT1	Oncogene	Cell viability, proliferation, apoptosis, and cisplatin sensitivity	miR-152-3p, HDGF, PI3K, and AKT	^180^
	Colorectal cancer	circ0001313	Oncogene	Cell proliferation and apoptosis	miR-510-5p, PI3K, and AKT2	^181^
	Colorectal cancer	circCDYL	Tumor suppressor	Cell viability, migration, invasion, and apoptosis	miR-105-5p, PTEN, PI3K, AKT, JAK2, and STAT5	^182^
	Colorectal cancer	circ0008285	Tumor suppressor		miR-382-5p, PTEN, PI3K, and AKT	^183^
	Liver cancer	circCDK13	Tumor suppressor	Cell migration, invasion, and cell cycle	JAK, STAT, PI3K, and AKT; tumor progression	^184^
	Liver cancer	circIGF1R	Oncogene	Cell proliferation, apoptosis, and cell cycle	PI3K, and AKT	^185^
	Liver cancer	circ0072309	Tumor suppressor	Cell viability, colony formation, invasion, and migration	miR-665, PI3K, AKT, Wnt, and β-catenin	^186^
	Liver cancer	circ0079299	Tumor suppressor	Tumor growth, cell cycle	PI3K, AKT, and mTOR; tumor size and tumor weight	^187^
	Liver cancer	circ0004001	Oncogene		miRNAs, VEGF, VEGFR, PI3K, AKT, mTOR, and Wnt	^188^
	Liver cancer	circ0004123	Oncogene		miRNAs, VEGF, VEGFR, PI3K, AKT, mTOR, and Wnt	^188^
	Liver cancer	circ0075792	Oncogene		miRNAs, VEGF, VEGFR, PI3K, AKT, mTOR, and Wnt	^188^
	Liver cancer	circEPHB4	Tumor suppressor	Cell viability, apoptosis, migration, and invasion	HIF-1α, PI3K-AKT, and ZEB1; tumor weight, tumor size, and metastasis foci	^189^
	Liver cancer	circCDYL	Oncogene		miR-892a, miR-328-3p, HDGF, HIF1AN, NCL, PI3K, AKT, NOTCH2, C-MYC, and SURVIVIN	^190^
	Hepatoblastoma	circHMGCS1	Oncogene	Cell proliferation, apoptosis, and glutaminolysis	miR-503-5p, IGF2, IGF1R, PI3K, and AKT	^193^
	Pancreatic cancer	circNFIB1	Tumor suppressor		miR-486-5p, PIK3R1, and VEGF-C	^194^
	Pancreatic cancer	circEIF6	Oncogene	Cell proliferation, migration, invasion, and apoptosis	miR-557, SLC7A11, PI3K, and AKT; tumor weight and volume	^195^
	Pancreatic cancer	circBFAR	Oncogene		miR-34b-5p, MET, and AKT; tumor weight and volume, Ki-67 level, MET inhibitor	^196^
Nervous system neoplasms	Glioma	circ0014359	Oncogene	Cell viability, migration, invasion, and apoptosis	miR-153, PI3K, and AKT	^197^
	Glioma	circDICER1	Oncogene	Angiogenesis	MOV10, miR-103a-3p, miR-382-5p, ZIC4, Hsp90β, PI3K, and AKT	^198^
	Glioma	circHIPK3	Oncogene	Cell proliferation, metastasis, apoptosis, and TMZ sensitivity	miR-524-5p, KIF2A, PI3K, and AKT; tumor growth	^199^
	Glioma	circPIP5K1A	Oncogene	Cell proliferation, invasion, apoptosis, and EMT	miR-515-5p, TCF12, PI3K, and AKT; tumor growth	^200^
	Glioma	circ104075	Oncogene	Cell proliferation, apoptosis, and autophagy	Wnt, β-catenin, PI3K, and AKT, il-104075, and Bcl-9	^201^
	Glioma	circ0000215	Oncogene	Cell proliferation, invasion, apoptosis, and EMT	miR-495-3p, CXCR2, PI3K, and AKT	^202^
	Glioblastoma	circAKT3	Tumor suppressor	Cell proliferation, and radiation resistance	PDK1, PI3K, and AKT; tumorigenicity	^62^
	Glioblastoma	circ0067934	Oncogene	Cell proliferation, metastasis, apoptosis, and EMT	PI3K and AKT	^206^
	Glioblastoma	circPVT1	Oncogene	Cell viability, migration, apoptosis, and EMT	miR-199a-5p, YAP1, PI3K, and AKT	^207^
	Neuroblastoma	circ0002343		EMT	RAC1, PI3K, AKT, and mTOR	^211^
Genitourinary tumors	Kidney cancer	circ0072309	Tumor suppressor	Cell proliferation, migration, invasion, and apoptosis	miR-100, PI3K, AKT, and mTOR	^218^
	Kidney cancer	circC3P1	Tumor suppressor	Cell viability, migration, invasion, and apoptosis	miR‐21, PTEN, PI3K, AKT, and NF‐κB	^219^
	Bladder cancer	circZNF139	Oncogene	Cell proliferation, migration, invasion, and cell clones		^220^
	Prostate cancer	circ0001085		EMT	miR-196b-5p, miR-451a, PI3K, and AKT	^228^
	Prostate cancer	circMBOAT2	Oncogene	Cell proliferation, migration, and invasion	miR-1271-5p, mTOR, PI3K, and AKT; tumor volume, tumor weight, Ki-67 expression, and mTOR	^227^
	Prostate cancer	circITCH	Tumor suppressor	Cell proliferation, migration, and invasion	Wnt, β-catenin, PI3K, AKT, and mTOR	^226^
	Prostate cancer	circNOLC1	Oncogene	Cell proliferation, and migration	NF-kappaB, miR-647, PAQR4, PI3K, and AKT	^225^
	Ovarian cancer	circRHOBTB3	Tumor suppressor	Cell proliferation, metastasis, and glycolysis	PI3K and AKT	^231^
	Endometrial cancer	circ0002577	Oncogene	Cell proliferation, migration, and invasion	miR-625-5P, IGF1R, PI3K, and AKT; tumor growth, and metastasis	^232^
	Cervical cancer	circCSPP1	Oncogene	Cell proliferation and migration	miR-361-5p, ITGB1, PI3K, and AKT	^233^
Tumors of the endocrine system	Thyroid cancer	circ0067934	Oncogene	Cell proliferation, migration, invasion, apoptosis, and EMT	PI3K and AKT	^238^
	Thyroid cancer	circ0007694	Tumor suppressor	Cell proliferation, migration, invasion, and apoptosis	PI3K, AKT, mTOR, and Wnt; tumor growth	^239^
	Thyroid cancer	circpsd3	Oncogene	Cell proliferation, metastasis, apoptosis, and cell cycle	miR-637, HEMGN, PI3K, and AKT	^240^
Tumors of the respiratory system	Lung cancer	circGFRA1	Oncogene		miR-188-3p, PI3K, and AKT; cell proliferation	^245^
	Lung cancer	circ100876		Cell proliferation and apoptosis	miR-636, RET, PI3K, and AKT	^247^
	Lung cancer	circ0018818	Oncogene	Cell proliferation, invasion, apoptosis, and EMT	miR-767-3p, Nidogen 1(NID1), PI3K, and AKT	^246^
Tumors of the musculoskeletal system	Osteosarcoma	circ0001785	Oncogene	Cell proliferation and apoptosis	miR-1200, HOXB2, PI3K, AKT, and Bcl-2	^250^
	Osteosarcoma	circEIF4G2	Oncogene	Cell proliferation, migration, and invasion	miR-218, PI3K, and AKT	^251^
	Osteosarcoma	circITCH	Tumor suppressor	Cell viability, proliferation, migration, invasion, and apoptosis	miR-22, PTEN, SP-1, PI3K, and AKT	^252^
	Osteosarcoma	circ0005909	Oncogene	Cell viability and cell clones	miR-338-3p, HMGA1, MAPK-ERK, PI3K, and AKT	^253^
Tumors of other systems	Oral squamous cell carcinoma	circ043621	Oncogene	Cell proliferation, apoptosis, and cell cycle	MAPK, PI3K, AKT, and Bcl-2	^257^
	Oral squamous cell carcinoma	circ102459	Tumor suppressor	Cell proliferation, apoptosis, and cell cycle	MAPK, PI3K, AKT, and Bcl-2	^257^
	Multiple myeloma	circ0007841			miR-338-3p, BRD4, PI3K, and AKT	^261^
	Breast cancer	circ103809	Oncogene	Cell proliferation, apoptosis, and cell cycle	PI3K and AKT	^262^
	Breast cancer	circPRMT5	Oncogene	Cell proliferation, apoptosis, and angiogenesis	miR-509-3p, TCF7L2, PI3K, and AKT	^263^
	Breast cancer	circHIPK3	Oncogene	Cell viability, proliferation, migration, and invasion	miR-193a, HMGB1, PI3K, and AKT	^264^
	Breast cancer	circ0000442	Tumor suppressor	Cell viability, colony formation, and cell cycle	miR-148b-3p, PTEN, PI3K, and AKT	^265^
	Breast cancer	circ001569	Oncogene	Cell growth and metastasis	PI3K and AKT	^266^
	Breast cancer	circ0000199	Oncogene	Cell proliferation, migration, invasion, chemo-sensitivity, and autophagy	miR-206, miR-613, PI3K, AKT, and mTOR	^267^

**Table 2:** Relationship between circRNA/PI3K/AKT axis and clinical features in cancer

Cancer type	CircRNA	Expression	Related features	Refs.
Bladder cancer	circZNF139	Upregulated	Disease-free survival	^220^
Liver cancer	circIGF1R	Upregulated	Tumor size	^185^
Liver cancer	circRNA0072309	Downregulated	5-year survival	^186^
Liver cancer	circ0004001, circ0004123, and circ0075792	Upregulated	TNM stage, and tumor size	^188^
Thyroid cancer	circ0067934	Upregulated	Survival period and AJCC stage	^238^
Glioma	circPIP5K1A	Upregulated	Survival time, tumor volume, and tumor stage	^200^
Glioblastoma	circ0067934	Upregulated	Disease-free survival and overall survival	^206^
Colorectal cancer	circ0008285	Downregulated	Lymph node metastasis, TNM stage, and tumor size	^183^
Oral squamous cell carcinoma	circ043621	Upregulated	Clinical stage, lymph node metastasis, and differentiation degree	^257^
Oral squamous cell carcinoma	circ102459	Downregulated	Clinical stage, lymph node metastasis, and differentiation degree	^257^
Prostate cancer	circMBOAT2	Upregulated	Gleason score, pathological T stage, and disease-free survival	^227^
Breast cancer	circPRMT5	Upregulated	Overall survival	^263^
Breast cancer	cirCHIPK3	Upregulated	Overall survival	^264^
Breast cancer	circ001569	Upregulated	Lymph node metastasis, pathological stage, and overall survival	^266^
Breast cancer	circ0000199	Upregulated	Tumor size, TNM stage, ki-67 level, and 3-year survival	^267^
Esophageal cancer	circLPAR3	Upregulated	Lymph node metastasis and TNM stage	^171^
Esophageal cancer	circVRK1	Downregulated	Overall survival	^172^
Gastric cancer	circ0010882	Upregulated	Tumor size, histological grade, and overall survival	^176^
Gastric cancer	circ0023409	Upregulated	Tumor size, histological grade, and lymph nodes metastasis	^177^
Gastric cancer	ciRS-7	Upregulated	Overall survival	^178^
Pancreatic cancer	circNFIB1	Downregulated	Lymph node metastasis	^194^
Pancreatic cancer	circBFAR	Upregulated	TNM stage, overall survival, and disease-free survival	^196^
Endometrial cancer	circ0002577	Upregulated	Overall survival, histological grade, lymph node metastasis, and lymph vascular space invasion	^232^

## Digestive system neoplasms

### Esophageal cancer

The expression of circVRK1 and circLARP4 is significantly downregulated and circLPAR3 levels are increased in esophageal squamous cell carcinoma (ESCC).^171–173^ Low circVRK1 expression predicts poor overall survival in patients with ESCC.^172^ Elevated circLPAR3 levels are markedly associated with lymph node metastasis (LNM) and advanced TNM stage.^171^ In addition, researchers have also observed alterations in biological functions of the circRNA/PI3K/AKT axis by in vitro functional assays. Silencing of the circRNA cZNF292 inhibits the activity of tumor cells and promotes cell apoptosis in ESCC.^174^ Upregulation of circVRK1 suppresses cell proliferation, increases the radiosensitivity of ESCC cells, and attenuates epithelial–mesenchymal transition (EMT).^172^ CircLARP4 inhibits cell apoptosis and promotes cell proliferation in ESCC.^173^ Furthermore, cZNF292, circVRK1, and circLARP4 all inhibit ESCC cell migration. Contrary to the aforementioned investigations, circLPAR3 functions as a tumor oncogene and enhances the malignant phenotype of ESCC tumors.^171^ Mechanistically, circLPAR3 increases the expression of the MET gene to enhance the RAS/MAPK and PI3K/Akt pathways by sponging miR-198 in ESCC. Knockdown of cZNF292 induces inactivation of the PI3K/AKT pathway and upregulation of AMPK signaling to exert effects in ESCC.^174^ CircVRK1 functions as a tumor suppressor gene by upregulating PTEN and inhibiting the PI3K/AKT axis.^172^ Similarly, circLARP4 promotes the expression of PTEN and inactivates the PI3K/AKT pathway to suppress the progression of ESCC.^173^

### Gastric cancer

PI3K/AKT pathway-related circRNAs (circPIP5K1A, circ0010882, circ0023409, ciRS-7, circMAN2B2, and circPVT1) are all obviously upregulated in gastric cancer.^175–180^ The levels of circ0010882 and circ0023409 are positively associated with tumor size and histological grade in gastric cancer patients.^176,177^ In addition, higher expression of circ0010882 or ciRS-7 is associated with shorter overall survival. Circ0023409 promotes LNM in gastric cancer. In terms of biological function, increased circPIP5K1A, circ0010882, and circ0023409 expression reduces gastric cancer cell proliferation, migration, and invasion.^175–177^ High expression of circPVT1 may enhance the sensitivity of gastric cancer cells to cisplatin (DDP).^180^ We also found that circMAN2B2 upregulates cell viability and the surviving cell fraction by cell transfection experiments.^179^ Silencing of circ0010882 attenuated gastric cancer cell growth and motility in vitro.^176^ In terms of the mechanism, circPIP5K1A sponges miR-671-5p to facilitate tumor progression by upregulating the KRT80 and PI3K/AKT pathways in gastric cancer.^175^ Circ0010882 regulates biological functions by promoting PI3K/AKT/mTOR signaling.^176^ Further studies have demonstrated that circ0023409, ciRS-7, circMAN2B2, and circPVT1 regulate the PI3K/AKT pathway by acting as sponges of miRNAs in gastric cancer.^177–180^ For example, circ0023409 activates the PI3K/AKT pathway by sponging miR-542-3p to increase IRS4 levels.^177^ In addition, researchers have established in vivo xenograft nude mouse models to further explore the relationship between gastric cancer and the circRNA/PI3K/AKT axis. The expression of circPIP5K1A facilitates tumor growth in gastric cancer in vivo.^175^

### Colorectal cancer (CRC)

The expression level of circ0001313 is dramatically upregulated while the levels of circCDYL and circ0008285 are decreased in CRC.^181–183^ Circ0008285 expression is positively associated with LNM, tumor-node-metastasis (TNM) stage, and tumor size in patients with CRC.^183^ Functionally, circCDYL inhibits CRC cell migration and invasion.^182^ Circ0001313 and circCDYL significantly reduce cell apoptosis in CRC.^181,182^ Silencing the expression of circ0008285 enhances cell proliferation and migration in CRC.^183^ The expression of circ0001313 increases the level of AKT2, thus contributing to CRC progression by downregulating miR-510-5p expression.^181^ CircCDYL inactivates PI3K/AKT and JAK/STAT signaling by decreasing miR-150-5p levels in colon cancer.^182^ Circ0008285 expression reduces migration and proliferation via regulation of the miR-382-5p/PTEN/PI3K/AKT axis in CRC.^183^

### Liver cancer

A series of circRNAs related to the circRNA/PI3K/AKT axis has been found to be closely related to the occurrence and progression of hepatocellular carcinoma (HCC). These circRNAs with aberrant expression are listed in Table 1.^184–190^ Tumor size positively correlates with the expression of circIGF1R, circ0004001, circ0004123, and circ0075792 in HCC.^185,188^ High expression of circ0072309 is related to better 5-year survival in patients with HCC.^186^ Decreased circCDK13 levels enhance cell motility while low levels of circIGF1R inhibit cell growth in HCC.^184,185^ High expression of circ0072309 impairs cell growth and motility, affecting cell viability, colony formation, invasion, and migration.^186^ Mechanistically, circCDK13 inhibits HCC progression by regulating the PI3K/AKT and JAK/STAT pathways (Table 1).^184^ Circ0072309 functions as a sponge of miR-665 to negatively regulate the PI3K/AKT and Wnt/β-catenin pathways in the pathophysiologic processes of HCC.^186^ The expression of circEPHB4 impedes HCC progression by negatively regulating the HIF-1α/PI3K/AKT axis and HIF-1α/ZEB1 pathway.^189^ Hepatoblastoma is the most common primary malignant hepatic tumor in children.^191,192^ The expression of circHMGCS1 is significantly upregulated in hepatoblastoma cell lines compared to normal hepatocyte cells and HCC cells.^193^ circHMGCS1 also promotes cell proliferation and inhibits apoptosis in hepatoblastoma cell lines. CircHMGCS1 markedly upregulates the IGF2/IGF1R/PI3K/AKT axis to regulate proliferation by sponging miR-503-5p.^193^ The expression of circEPHB4 was negatively associated with tumor weight, size, and metastatic foci in vivo.^189^ A higher level of circ0079929 predicted decreased tumor size and weight in nude mouse models.^187^ CircCDK13 is an important negative regulator in the development and progression of HCC.^184^

### Pancreatic cancer

The level of circNFIB1 is markedly decreased while circEIF6 and circBFAR expression levels are elevated in pancreatic cancer.^194–196^ High expression of circNFIB1 restrains lymphatic metastasis of pancreatic cancer.^194^ Upregulated levels of circBFAR predict high TNM stage and poor prognosis.^196^ Functionally, we found that the expression of circEIF6 promotes cell proliferation, increases cell migration and invasion, and inhibits cell apoptosis by performing siRNA-mediated knockdown experiments in pancreatic cancer cells.^195^ Mechanistically, circNFIB1 induces VEGF-C inhibition and attenuates LNM by sponging miR-486-5p and inhibiting the PI3K/AKT pathway in pancreatic ductal adenocarcinoma.^194^ CircEIF6 regulates biological functions by upregulating miR-557 expression, downregulating SLC7A11 levels, and inactivating the PI3K/AKT pathway in pancreatic cancer.^195^ CircBFAR facilitates mesenchymal–epithelial transition by sponging miR-34b-5p and upregulating the MET/PI3K/AKT axis in pancreatic ductal adenocarcinoma.^196^ In vivo experiments showed that downregulation of circBFAR or circEIF6 expression can lead to lower tumor weight and volume in pancreatic ductal adenocarcinoma.^195^

## Nervous system neoplasms

### Glioma

**Fig. 3:**
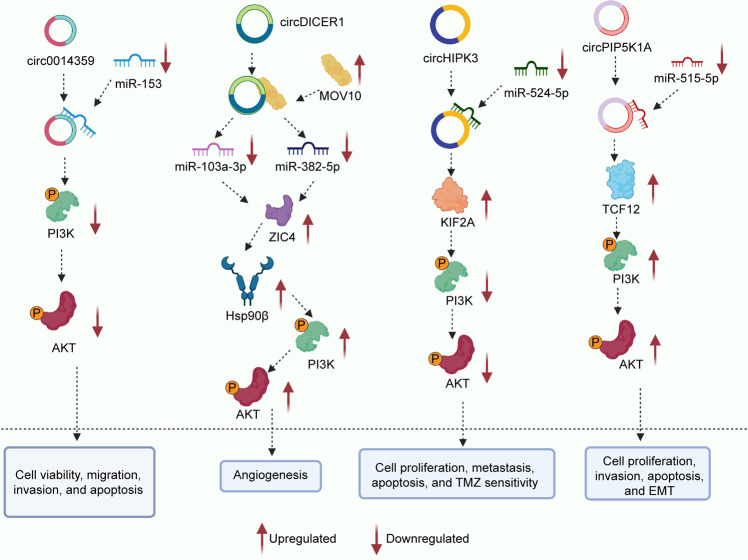
The specific mechanism of glioma progression between circRNAs and PI3K/AKT pathway. Circ0014359 exerts its effects by inhibiting the level of miR-153 and regulating the PI3K/AKT axis. CircDICER1 in combination with MOV10 plays a critical role in glioma angiogenesis via regulation of miR-103a-3p (miR-382-5p)/ZIC4. CircHIPK3 regulates biological functions to improve sensitivity to temozolomide through suppression of the miR-524-5p/KIF2A-mediated PI3K/AKT pathway. circRNAs can also facilitate glioma tumorigenesis and progression by regulating the circPIP5K1A/miR-515-5p/TCF12/PI3K/AKT axis in glioma. Image created with BioRender (https://biorender.com/)

PI3K/AKT axis-associated circRNAs are significantly upregulated in glioma (Table 1).^197–202^ Elevated circPIP5K1A expression is positively correlated with shorter survival time, larger tumor volume, and higher tumor stage in patients with glioma.^200^ CircHIPK3, circPIP5K1A, circ104075, and circ0000215 increase glioma cell proliferation in vitro.^197,199,200,202^ Cic0014359, circHIPK3, circPIP5K1A, and circ0000215 facilitate cell motility in glioma.^197,199,200,202^ Furthermore, circDICER1 markedly attenuates the angiogenesis of glioma-exposed endothelial cells.^198^ Downregulated expression of circHIPK3 induces a significant upregulation of temozolomide sensitivity in glioma.^199^ Mechanistic studies have revealed that circ0014359 exerts its effects by inhibiting the level of miR-153 and regulating the PI3K axis in glioma^197^ (Fig. 3). CircDICER1 in combination with MOV10 plays a critical role in glioma angiogenesis via regulation of miR-103a-3p (miR-382-5p)/ZIC4.^198^ CircHIPK3 regulates biological functions to improve sensitivity to temozolomide through suppression of the miR-524-5p/KIF2A-mediated PI3K/AKT pathway.^199^

A series of studies have shown that circRNAs can facilitate glioma tumorigenesis and progression by regulating the circPIP5K1A/miR-515-5p/TCF12/PI3K/AKT and circ0000215/miR-495-3p/CXCR2/PI3K/AKT pathways^200,202^ (Fig. 3). Glioblastoma (GBM) is the most malignant glioma and has an extremely poor prognosis.^203–205^ CircAKT3 is overexpressed while circ0067934 and circPVT1 expression are significantly downregulated in GBM.^62,206,207^ A higher level of circ0067934 portends shorter disease-free survival and decreased overall survival rates in GBM.^206^ Inhibition of circ0067934 expression may be a promising strategy for improving GBM prognosis. The upregulation of circAKT3 suppresses GBM cell proliferation and increases sensitivity to radiation.^62^ The expression of circ0067934 facilitates cell proliferation and metastasis and inhibits cell apoptosis in GBM by upregulating the PI3K-AKT pathway.^206^

### Neuroblastoma (NB) and pituitary tumor

NB is the most common extracranial solid tumor in childhood.^208–210^ The expression of circ0002343 was found to be involved in the regulation of EMT in NB.^211^ circ0002343 significantly affects EMT by regulating the RAC1/PI3K/AKT/mTOR axis. Pituitary tumors are some of the most common benign neoplasms of the central nervous system.^212,213^ The levels of circ0054722, circ0012346, and circ0007362 are significantly increased while the expression of some circRNAs (circ0062222, circ0016403, circ0033349, and circ0049730) is downregulated in invasive nonfunctioning pituitary adenomas compared with the levels in noninvasive nonfunctioning pituitary adenomas.^214^

### Genitourinary tumors

#### Kidney cancer and bladder cancer

**Fig. 4:**
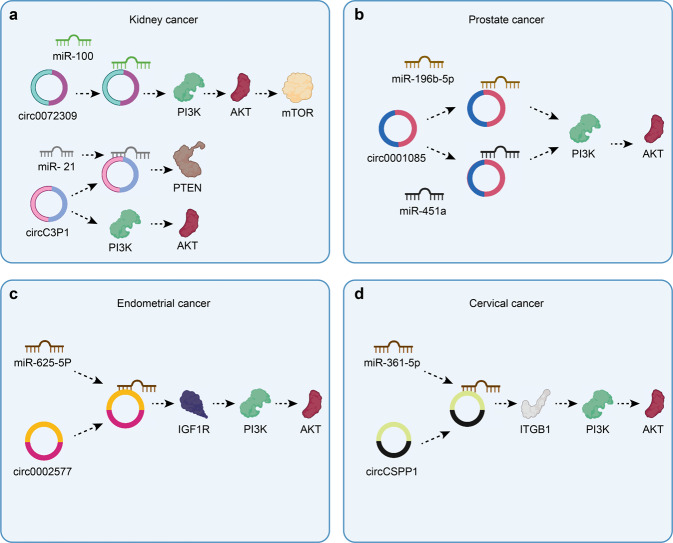
The specific mechanism of circRNAs and PI3K/AKT pathway in different cancers. **a** Circ-0072309 sponges miR-100 to inhibit the PI3K/AKT/mTOR pathway in kidney cancer. CircC3P1 inhibits kidney cancer progression via regulation of miR/PTEN pathways and the PI3K/AKT pathway. **b** Circ0001085 regulates prostate cancer progression through the PI3K/AKT pathway by sponging miR-196b-5p and miR-451a. **c** Overexpression of circ0002577 enhances the IGF1R/PI3K/AKT axis to increase the migration, invasion, and proliferation of endometrial cancer cells. **d** CircCSPP1 expression inhibits cervical cancer cell apoptosis and promotes cell proliferation and migration via the miR-361-5p/ITGB1/PI3K/AKT axis in cervical cancer. Image created with BioRender (https://biorender.com/)

Kidney cancer is not a single disease but comprises different types of cancer that occur in the kidney.^215–217^ Renal carcinoma-associated transcripts (circ0072309 and circC3P1) are significantly downregulated in renal carcinoma tissues compared to corresponding normal tissues.^218,219^ These circRNAs significantly suppresses cell proliferation, migration, and invasion and promote cell apoptosis in kidney cancer. Circ-0072309 sponges miR-100 to inhibit the PI3K/AKT and mTOR pathways in kidney cancer.^218^ CircC3P1 exerts diverse biological functions by inhibiting the PI3K/AKT and NF-κB pathways by regulating the miR-21/PTEN axis^219^ (Fig. 4a). The overexpression of circZNF139 is markedly associated with disease-free survival in bladder cancer.^220^ circZNF139 overexpression also attenuates bladder cancer cell proliferation, colony formation, migration, and invasion by regulating the PI3K/AKT pathway.

#### Prostate cancer (PCa)

PCa is a major cause of male cancer-related mortality worldwide.^221–224^ The level of circNOLC1 is increased while circITCH expression is obviously downregulated in PCa.^225,226^ CircMBOAT2 is overexpressed in PCa and contributes to poor prognosis.^227^ Moreover, increased circMBOAT2 levels are positively correlated with Gleason score and pathological T stage. Functionally, circNOLC1, circITCH, and circMBOAT2 govern multiple cellular processes, such as cell proliferation, migration, and invasion, via the circRNA/PI3K/AKT axis in PCa.^225–227^ Circ0001085 induces EMT in PCa cells in vitro.^228^ Circ0001085 regulates PCa progression through the PI3K/AKT pathway by sponging miR-196b-5p and miR-451a (Fig. 4b). CircMBOAT2 clearly promotes tumorigenesis and metastasis in PCa in vivo.^227^

#### Female reproductive system cancers

Ovarian, endometrial, and cervical cancer are three major malignant tumors causing a severe threat to women’s health.^229,230^ The downregulation of circRHOBTB3 not only attenuates cell proliferation and metastasis but also inhibits glycolysis by suppressing the PI3K/AKT pathway in ovarian cancer.^231^ Circ0002577 expression is markedly increased in endometrial cancer.^232^ Circ0002577 expression is positively correlated with the histological grade of the tumor, LNM, and lymph vascular space invasion. Studies have revealed that patients with high expression of circ0002577 have a poor prognosis. The overexpression of circ0002577 enhances the IGF1R/PI3K/AKT axis to increase the migration, invasion, and proliferation of endometrial cancer cells (Fig. 4c). Silencing of circ0002577 expression significantly inhibits the growth and metastasis of tumors in nude mouse models of endometrial cancer.^232^ The expression of circCSPP1 is markedly upregulated in cervical cancer tissues.^233^ CircCSPP1 expression inhibits cervical cancer cell apoptosis and promotes cell proliferation and migration via the miR-361-5p/ITGB1/PI3K/AKT axis in cervical cancer (Fig. 4d).

## Tumors of the endocrine system

Thyroid cancer is the most common malignancy occurring in the endocrine system.^234–237^ The expression of circ0067934 and circpsd3 is upregulated whereas circ0007694 expression is downregulated in thyroid tumors.^238–240^ High circ0067934 expression is associated with a shorter survival period of thyroid cancer patients.^238^ The expression of circ0067934 and circ0007694 affects diverse cell biological functions, such as cell proliferation, migration, invasion, and apoptosis, in thyroid cancer via the PI3K/AKT signaling pathway.^238,239^ During the regulation of different cellular biological processes, circ0067934 acts as an oncogene, but circ0007694 may function as a tumor suppressor gene in the progression of thyroid cancer. Increased circ0007694 expression effectively suppresses the growth of papillary thyroid carcinoma in vivo.^239^

## Tumors of the respiratory and musculoskeletal systems

### Lung cancer

**Fig. 5:**
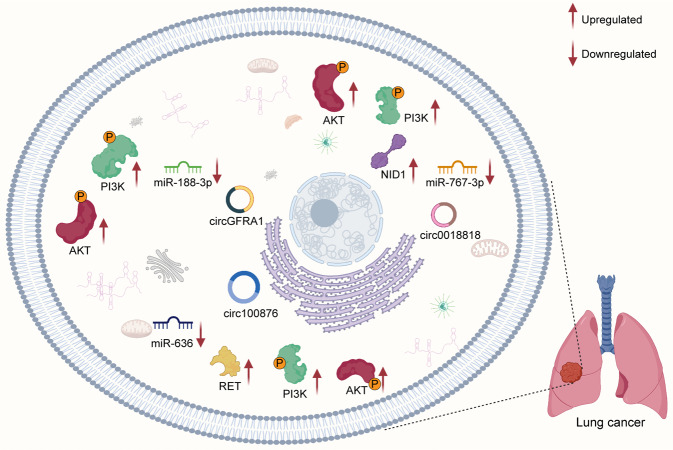
The mechanism of circRNAs and PI3K/AKT pathway in lung cancer. CircGFRA1 activates the PI3K/AKT pathway by downregulating the expression of miR-188-3p in lung cancer. Circ100876 affects biological functions via PI3K/AKT signaling by regulating the miR-636/RET axis. The circ0018818/miR-767-3p/NID1/PI3K/AKT axis also plays a key role in the progression of lung cancer. Image created with BioRender (https://biorender.com/)

Lung cancer is one of the leading causes of cancer-related death worldwide, with NSCLC accounting for 85% of all lung cancers.^241–244^ The expression of circGFRA1 and circ0018818 is significantly upregulated in NSCLC tissues compared to normal counterparts.^245,246^ Silencing of circ0018818 expression inhibits proliferation, invasion, and EMT and promotes cell apoptosis.^246^ In addition, circGFRA1 activates the PI3K/AKT pathway by downregulating the expression of miR-188-3p in lung cancer. Knockdown of circ100876 reduces cell proliferation, migration, and invasion and facilitates NSCLC cell apoptosis by regulating the miR-636/RET axis and PI3K/AKT signaling.^247^ The circ0018818/miR-767-3p/NID1/PI3K/AKT axis also plays a key role in the progression of lung cancer (Fig. 5).

#### Osteosarcoma (OS)

OS is the most common primary malignant bone tumor in children and adolescents.^248,249^ The expression of circRNAs associated with the PI3K/AKT axis is listed in Table 1.^250–253^ The expression of circEIF4G2 and circITCH affects cell biological functions, such as cell proliferation, migration, and invasion, in OS.^251,252^ Silencing of circ0005909 obviously decreases cell viability and cell clone capacity in OS cell lines.^253^ Decreased expression of circ0001785 reduces cell proliferation and facilitates cell apoptosis in OS.^250^ Mechanistically, the expression of circ-ITCH attenuates cell biological functions because circ-ITCH acts as a competing endogenous RNA (ceRNA) for miR-22 to inactivate the PTEN/PI3K/AKT and SP-1 pathways in OS.^252^ Circ0005909 expression enhances OS malignant progression by upregulating the MAPK-ERK and PI3K-Akt signaling pathways by sponging miR-338-3p to inhibit the level of HGMA1.^253^

### Tumors of other systems

Oral squamous cell carcinoma (OSCC) is a malignant type of head and neck squamous cell carcinoma.^254–256^ Circ043621 expression is remarkably elevated and circ102459 levels are dramatically decreased in OSCC tissues.^256^ CircPARD3 and circ043621 expression levels are relatively associated with clinical stage, LNM, and differentiation degree in OSCC. In vitro assays have revealed that increased circ043621 levels and decreased circ102459 expression can induce arrest in the G0 and/or G1 phase, apoptosis, and inhibition of cell proliferation by activating the MAPK and PI3K/AKT pathways.^257^ Multiple myeloma (MM) is a plasma cell malignancy.^258–260^ The expression of circ0007841 is significantly upregulated in MM cell lines and bone marrow-derived cells.^261^ High circ0007841 expression enhances the malignant behaviors of MM cells, for example, promoting cell proliferation, cell cycle progression, and metastasis, by activating the PI3K/AKT pathway.

PI3K/AKT axis-associated circRNAs are aberrantly regulated in breast cancer^262–267^ (Table 1). The overexpression of circ0000199 is significantly associated with tumor size, TNM stage, and Ki-67 level in patients with breast cancer.^267^ Higher levels of circPRMT5, circHIPK3, circ001569, and circ0000199 predict poor prognosis in breast cancer.^263,264,266,267^ circ0000199 can affect tumor cell tolerance of chemotherapy via suppression of the PI3K/AKT/mTOR pathway and activation of the miR-206/miR-613 axis.^267^ circ0000199 also enhances cell proliferation, migration, and invasion in breast cancer. Silencing of circPRMT5 expression attenuates angiogenesis and proliferation and induces apoptosis.^263^ CircPRMT5 contributes to malignant phenotypes by activating the PI3K/AKT/mTOR axis via regulation of the miR-509-3p/TCF7L2 pathway. High expression of cirCHIPK3 significantly promotes cell migration, invasion, viability, and proliferation by targeting the miR-193a/HMGB1/PI3K/AKT axis.^264^ High circ0000442 expression induces suppression of cell viability and cell cycle arrest at the G1 phase and decreases colony formation in breast cancer.^265^ circ0000442 knockdown experiments have further confirmed this result. circ0000442 acts as a sponge of miR-148b-3p to downregulate the PTEN/PI3K/AKT pathway to impede tumor progression. Moreover, the knockdown of cirCHIPK3 attenuates breast cancer growth in vivo.^264^

### CircRNAs related to the PI3K/AKT pathway as biomarkers

In recent years, researchers have focused on identifying effective molecular biomarkers to improve the early detection, monitoring, and prediction of therapy response in cancer patients.^268–270^ Technological advances have contributed to an up-to-date understanding of the roles of circRNAs in the initiation and progression of cancer. A growing number of circRNAs related to the PI3K/AKT pathway have been found to be potential biomarkers for the diagnosis, treatment, and prognostication of many cancers. In this section, we will further discuss the important role of circRNAs in clinical applications.

### Diagnostic biomarkers

The diagnosis of cancer at an early stage is critical for effective treatment and monitoring.^271,272^ A critical factor of early diagnosis is the identification of diagnostic biomarkers.^273–275^ Many circRNAs in the PI3K/AKT pathway have been identified as aberrantly expressed during the progression of different cancers (Table 1). For example, the expression of circCSPP1 is markedly upregulated in cervical cancer tissues.^233^ The expression of circGFRA1 and circ0018818 is significantly upregulated in NSCLC tissues compared to normal tissues.^245,246^ CircRNAs with significantly abnormal expression have diagnostic potential in many cancers. In addition, the levels of circ0004001, circ0004123, and circ0075792 in serum are markedly upregulated in patients with HCC.^188^ The expression of circ0010882 in serum is obviously elevated in gastric cancer patients.^176^

The expression of circ0007841 in serum is significantly increased in MM patients.^261^ These results suggest that early diagnosis based on circRNAs is practical. More studies about the diagnostic roles of circRNAs in serum are needed.

### Prognosis prediction

Emerging evidence suggests that many circRNAs are reliable for predicting the prognosis of patients with cancer.^196,276,277^ which provides important guidance for cancer therapy. A significant number of circRNAs have been found to be markedly associated with survival parameters, such as overall survival, disease-free survival, and the 5-year survival rate (Table 2). Low circVRK1 expression predicts poor overall survival in patients with ESCC.^172^ A higher level of circ0067934 portends shorter disease-free survival and decreased overall survival rates in GBM.^206^ The expression of circ0072309 is positively correlated with the 5-year survival rate in patients with liver cancer.^186^ In addition, some circRNAs have been found to be significantly associated with other clinical features in cancer. Elevated circLPAR3 levels are markedly associated with LNM and advanced TNM stage in esophageal cancer.^171^ The levels of circ0010882 and circ0023409 are positively associated with tumor size and histological grade in gastric cancer patients.^176,177^ The elevated expression of circMBOAT2 has positively correlated with the Gleason score and pathological T stage in PCa.^227^ These results provide an important reference for cancer treatment.

### Targeted therapies

Targeted therapy, a recent trend in cancer therapy, is emerging as a novel therapeutic strategy.^278–280^ Targeted therapies significantly enhance the efficiency of cancer therapy.^281,282^ CircRNAs can positively or negatively modulate biological functions and cancer progression through multiple signaling pathways. CircLARP4 promotes the expression of PTEN and inactivates the PI3K/AKT pathway to suppress the progression of ESCC.^173^ CircPIP5K1A sponges miR-671-5p to facilitate tumor progression by upregulating KRT80 and the PI3K/AKT pathway in gastric cancer.^175^ Circ0067934 facilitates cell proliferation and metastasis and inhibits cell apoptosis in GBM by upregulating the PI3K-AKT pathway.^206^ CircEPHB4 impedes HCC progression by negatively regulating the HIF-1α/PI3K/AKT axis and the HIF-1α/ZEB1 pathway in HCC.^189^ Upregulating or downregulating the expression of circRNAs may be a feasible way to regulate tumor progression. Silencing of circ0010882 attenuates gastric cancer cell growth and motility in vitro.^176^ Knockdown of circ100876 reduces cell proliferation, migration, and invasion and facilitates NSCLC cell apoptosis.^247^ In addition, a miR-671-5p inhibitor was able to significantly reduce the level of circPIP5K1A to inhibit the progression of gastric cancer.^175^ Rapamycin, an mTOR inhibitor, blocks the circMBOAT2/PI3K/AKT/mTOR pathway to suppress PCa progression.^227^ CircHIPK3 regulates biological functions to improve sensitivity to temozolomide through suppression of the miR-524-5p/KIF2A-mediated PI3K/AKT pathway in glioma.^199^ Circ0000199 can make tumor cells sensitive to chemotherapy via suppression of the PI3K/AKT/mTOR pathway and activation of the miR-206/miR-613 axis in breast cancer.^267^ High expression of circPVT1 enhances the sensitivity of gastric cancer cells to cisplatin (DDP).^180^ These results provide important information for the clinical treatment of cancers.

## Conclusions and future perspectives

CircRNAs are emerging biomarkers in cancer diagnosis and treatment. Complex circRNA regulatory networks have important implications in cancer research and have revolutionized our views on cancer genesis, progression, and treatment. In terms of circRNA-mediated cellular signaling studies, the most exciting finding is that circRNAs can function through molecular associations with the components of classical signaling pathways. The PI3K/AKT pathway is closely associated with the pathogenesis and development of cancer. It can regulate cell survival and proliferation and plays an essential role in cell migration, invasion, and angiogenesis. The circRNA/PI3K/AKT axis has recently attracted increasing attention. The modulating effect of tumor cellular biological functions is of interest for researchers studying the circRNA/PI3K/AKT axis. In terms of the circRNA/PI3K/AKT axis, plenty of circRNAs have been extensively studied. The ubiquitous expression and tumor specificity of circRNAs have ushered in new opportunities for cancer diagnosis. The expression of circRNAs is significantly associated with the clinical phenotype and survival time, indicating that it has important guiding significance for cancer prognostic evaluation. However, the expression level and expression stability of circRNAs in circulating body fluids need further study. Assessment of the expression stability of circRNAs in circulating body fluids, including urine and blood, has vast prospects in terms of clinical applications. In addition, considering the aberrant expression of a large number of cancer-related circRNAs, it is crucial to identify circRNAs related to certain types of cancer.

CircRNAs positively or negatively regulate biological functions in cancer development and progression via the PI3K/AKT signaling pathway. Thus, we may control the cancer process by regulating circRNAs in the circRNA/PI3K/AKT axis. The implementation of this idea relies on in-depth research of pharmacologic therapies. A promising drug must stably regulate circRNA activity and efficiently transduce the effect, thus controlling cancer progression. This necessitates a deeper understanding of the functions and mechanisms of circRNA related to the PI3K/AKT pathway under physiological and pathophysiological conditions. At present, research on the circRNA/PI3K/AKT axis is still in its infancy. Structural and functional data for circRNAs related to PI3K/AKT pathway remain limited. The mechanism of interactions between circRNAs and the PI3K/AKT pathway has yet to be established. Without detailed information on the structure and function of circRNAs, therapeutic options based on PI3K/AKT pathway are difficult to identify.
